# The Venom of Spectacled Cobra (Elapidae:* Naja naja*):* In Vitro* Study from Distinct Geographical Origins in Sri Lanka

**DOI:** 10.1155/2018/7358472

**Published:** 2018-09-27

**Authors:** Duminda S. B. Dissanayake, Lasanthika D. Thewarage, Roshitha N. Waduge, J. G. S. Ranasinghe, S. A. M. Kularatne, R. P. V. Jayanthe Rajapakse

**Affiliations:** ^1^Department of Veterinary Pathobiology, Faculty of Veterinary Medicine and Animal Science, University of Peradeniya, Peradeniya, Sri Lanka; ^2^Department of Pathology, Faculty of Medicine, University of Peradeniya, Peradeniya, Sri Lanka; ^3^Department of Biochemistry, Faculty of Medicine, University of Peradeniya, Peradeniya, Sri Lanka; ^4^Department of Medicine, Faculty of Medicine, University of Peradeniya, Peradeniya, Sri Lanka

## Abstract

Several countries residing envenomation due to* Naja naja* had revealed a disparity in the venom composition according to their geographic location and Sri Lankan cobra still lacks the evidence to support this. Therefore, the current study was focused on addressing relationship between the histopathological changes according to geographic variation of Sri Lankan* N. naja* venom. The histopathological changes in vital organs and muscle tissues following intramuscular administration of venom of* N. naja* were studied using BALB/c mice. The median lethal dose of venom of* N. naja* in the present study was determined to be 0.55, 0.66, 0.68, 0.62, and 0.7 mg/kg for North (NRP), Central (CRP), Western, Southern, and Sabaragamuwa Regional Population venoms, respectively. Histopathological changes were observed in different levels in vital organs and muscle tissues of mice. NRP accompanied significantly higher infiltration of inflammatory and necrotic cells into skeletal muscle and CRP venom demonstrated high level of cardiotoxic effects comparing to other regions. This study revealed a certain extent of variations in the pathological effects of* N. naja *venom samples according to their geographical distribution.

## 1. Introduction

**Figure 1:**
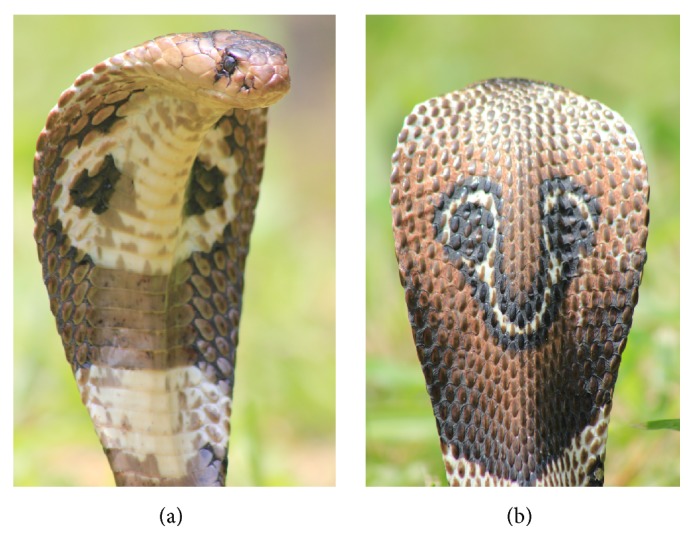
Spectacled cobra (*Naja naja*); (a) distinct hood and (b) spectacle pattern on the hood.

Many tropical and subtropical countries siding with the serious medical concern of envenomation report an approximate annual rate of 90000 accidental human deaths due to snakebites [1–3]. Being a tropical country with diverse climatic and geographical distribution, Sri Lanka provides an excellent natural habitat for both venomous and nonvenomous snakes and this contributes to demonstrating a significant approximate rate of 37000 snakebites cases [4, 5]. Most of the countries including Sri Lanka residing this topic of envenomation have categorized the venomous snakes responsible according to the severity of their toxicity. In this classification, Asian cobra is recognized as one of the highly venomous snakes. Though the frequency of cobra bites are less than viper bites in Sri Lanka, rates of mortality and morbidity in the cases of cobra envenomation are highly notable [6]. Asian cobra including* Naja naja* (Figure 1) species in Sri Lanka, which taxonomically belongs to family Elapidae, is well known for its envenomation cases due to its unique haemorrhagic, hemolytic, inflammatory, and necrotic effects on different organs leading to multiple organ failure [6]. Similar to many highly elaborated snake venom, cobra venom also comprises the major two fractions of toxic enzymatic proteins and nonenzymatic proteins also identified as cytotoxins [7, 8]. Hydrolytic enzymes of PLA_2_, hyaluronidase, caseinolytic AMPase, and ATPase when combined with cytotoxic neurotoxins, cobramines, membranotoxins, and cardiotoxins contribute progressively to exerting their main expected activity of immobilizing and digesting the venom inserted prey [9–11]. These inductions of toxic effects by the above biological active components generally attribute towards generating visible pathological anomalies on cardiac, nervous, renal, pulmonary, hepatic, and muscle tissues.

Pathological alterations induced by snakebite usually depend upon two basic factors: the species of snake responsible for the bite and the biochemical composition of the specific venom of that species [12–14]. Many scientists are using different methodologies and techniques to address this biochemical and pharmacological properties of snakes' venoms. Composition of the snake venoms varies according to their taxonomic differences [15–18], geography [16, 19–25], diet [26–30], age [31, 32], and prominent variations depending on the sex of the snake [26, 33]. Out of these factors, intra- and interspecies variation of venom composition due to geographical difference has become a controversial topic as the production of efficacious antivenom primarily relies on the composites of the venom.

India is inhabited with four species of cobras,* N. naja*,* N. kaouthia*,* N. sagittifera*, and* N. oxiana,* and out of these,* N. naja* is identified to be the most distributed species of* Naja* in India. Therefore, India has already given their much concern to address this issue of venom variability due to regional difference of* N. naja.* Several studies already have revealed that composition and clinicopathological effects on vital organs by venom of* N. naja* have a significant variation in accordance with their originated location [16–19, 34–37]. Similar to* N. naja* in India, the spectacled cobra in Sri Lanka inhabits a wide range of terrestrial habitat, up to an elevation of 1500m [38, 39]. Though the distribution of Sri Lankan* N. naja* is highly variable, Sri Lanka still lacks these systematics studies on Sri Lankan venomous snakes to verify each venom composition and their effect on body according to their geographic diversity and other associated factors. When overviewing the available literature on lethality and toxic effect of Sri Lankan* N. naja* venom on vital organs, the data records do not reveal sufficient evidence to support the factors of intraspecies variability due to location of origin.

Antivenom is the only specific, best-preferred, and worldwide selection of treatment for snakebites [3]. Sri Lanka still lacks the facility of producing antivenom for its native and endemic venomous snakes species including cobra [40]. Currently, treatments for the snakebite victims in Sri Lanka are carried out using polyvalent or bivalent antivenom, which is usually manufactured in India. Several studies on* in vitro* antivenom efficacy and other studies reveal that the current clinical management done using available antivenom as the treatment for the envenomation due to cobra bites in Sri Lanka does not trigger the desired level of efficacy in recovering the patient and this unveils the necessity of understanding the specific biological composition and their related pathogenesis in relation to their geographic location [6, 37, 40–42].

Therefore, primary objective of the present study was to identify the lethality and specific histopathological effects induced by the venom of* N. naja* in Sri Lanka on vital organs and muscle tissues using BALB/c mice models. This study also investigated the venom variability according to five distinct regions, considering the type and severity level of tissue injury as visible indicators of effect of some* N. naja* venom components. This acts as a fundamental study contributing in identification of interspecies qualitative and quantitative variation of venom of Sri Lankan* N. naja*, thereby supporting the future production of efficacious antivenom against* N. naja* venom.

## 2. Material and Methods

### 2.1. Snakes, Venom Collection, and Storage

**Figure 2:**
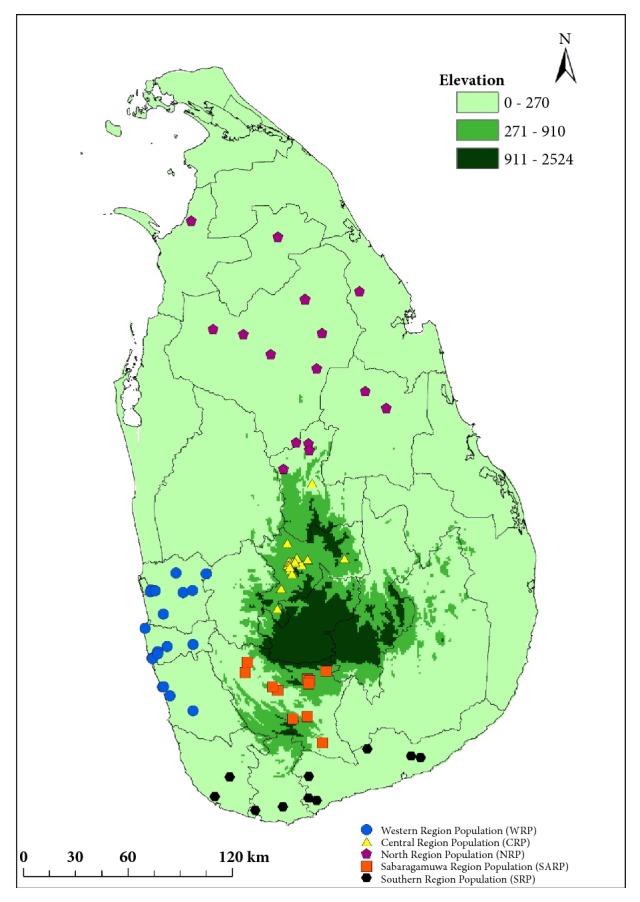
The schematic representation of the collection localities of Indian cobra venom samples.

Seventy-seven adult cobras were collected from five distinct geographic areas of mainland Sri Lanka varying from several geographic characters such as rainfall, forest type, altitude, and moisture [20 specimens from Western Region Population (WRP), 21 specimens from Central Region Population (CRP), 15 specimens from North Region Population (NRP), 11 specimens Sabaragamuwa Region Population (SARP), and 10 specimens from Southern Region Population (SRP)] (Figure 2) with in the period of March 2014 to end of April 2015. The snakes were transferred to the herpetarium, Department of Veterinary Pathobiology, Faculty of Veterinary Medicine and Animal Science, University of Peradeniya. The venom collection from each cobra was performed in the herpetarium by allowing the cobra to bite onto a parafilm stretched over a 50ml sterile beaker. The venom was immediately frozen at -80°C. These frozen venom samples were used within three months from the collection for each experiment.

### 2.2. Reagents and Animals

All the reagents and chemicals used in these studies were of analytical grade. Albino mice weighing 22-23g and the mice were obtained from Medical Research Institute (MRI), Colombo, and were housed in standard conditions at the small animal unit, Faculty of Medicine, University of Peradeniya, and fed with normal diet and water. The animals were handled according to the following guidelines given by Council for International Organizations of Medical Sciences on animal experimentation [43]. In order to gain sufficient adaptation to the new environment, these mice were housed week prior to the beginning of all experiments.

### 2.3. Estimation of Protein

The pooled venom samples of each region were directly quantified in order to standardize each venom sample from each region. Standardization was done using Bradford protein assay method [44]. The total protein concentration was estimated at 595 nm in 96 well-microplate reader (Thermo Multiskan Ex, USA) according to manufacturer's protocol. A blank was prepared by using 10*μ*l of distilled water. All standards and samples were in triplicate. The corresponding protein concentrations were estimated using absorbance curve of standard dilutions. All doses of venom mentioned in this paper, which referred to respective venom protein doses, have been expressed in mg/Kg.

### 2.4. Lethality Assay (LD_50_)

As the first step of assessing correct lethal doses, cobra venom was injected intramuscularly (IM) into right gastrocnemius of mice. This pilot study venom doses from each sample ranged from 0.2mg/Kg to 1.2mg/Kg and was prepared in total volume of 500*μ*l. According to the results achieved from the above first study general median value on LD_50_ of each geographically pooled venom sample was identified. Then ten doses ranging around this temporary median LD_50_ value were prepared for each venom sample. Six mice per each identified venom dose were given with IM injection. Number of mice that died within 24 hours of envenoming in each test group was recorded. A control group of 10 mice were injected with a similar volume of the diluent. The LD_50_ values were then calculated using probit analysis method [45] using IBM SPSS Statistics for Windows, Version 17.0.

### 2.5. Histopathological Assay

For assessment of histopathological studies on vital organs, five groups of mice (12 mice for each group) were injected with intramuscular injection of same tested dose (0.5mg/kg) of venom from each geographically separated sample. The control groups of mice were injected with sterile 0.9% NaCl solution in volume of 500ml into the same site where tested group received the venom injection. After that autopsies were performed at 1h, 3h, and 6h following administration of cobra venom into mice. Three mice from each treatment group were euthanized and then tissue samples from venom injected site of right gastrocnemius muscle, kidneys, liver, heart, lung, and spleen were collected and these samples were preserved in 10% neutral buffered formalin for at least 7 days. After that for histopathological preparation samples were dehydrated, embedded in wax, and 4 to 5*μ*m paraffin sections were cut and stained with hematoxylin and eosin [46]. The stained preparations were examined using Axio Scope. A1 (ZEISS) light microcopy at a magnification of 40x and micrographs were taken using a digital camera unit. Histopathological damage on each tissue sample was scored as follows: absent, 0; mild, +; moderate, ++; and severe, +++ [47].

### 2.6. Serum Glutamate Oxaloacetate Transaminase (SGOT), Serum Glutamate Pyruvate Transaminase (SGPT), and Serum Creatine Kinase (CK) Activities

For the assessment of anomalies in SGOT, SGPT, and serum creatinine blood was collected via intracardiac method. Blood collection was also performed at 1h, 3h, and 6h following administration of cobra venom into mice and three mice from each treatment group were sacrificed to fulfill this purpose. The activities of serum glutamate oxaloacetate transaminase (SGOT) and serum glutamate pyruvate transaminase (SGPT) were measured by using commercial available kits. Methods described in the kit followed [48]. Activities of enzymes were expressed as U/L. The enzymatic activity of Serum Creatine Kinase (CK) content was estimated according to the method which is described in the commercially available diagnostic kit [49]. One-way ANOVA was used to test the relationship between pooled venom samples from each geographic region and each enzymatic activity using SPSS (17.0). Results were considered to be significant at p < 0.05.

## 3. Results

### 3.1. Snakes

We collected 77* N. naja *according to the geographic distinct localities in Sri Lanka (Figure 2).

### 3.2. Control Group

Mice were injected with 500 *μ*l doses of sterile 0.9% NaCl solution in the gastrocnemius muscle and sacrificed after 1 hr, 3 hr, 6 hr, and one week. Normal histological appearance was observed in all the organs (kidneys, livers, hearts, lungs, and spleens) except in gastrocnemius muscle tissue fiber, which contained monofocal lesions due to the needle inserted to inject saline. There was no inflammatory infiltrate or fibrosis.

### 3.3. Lethality of the Venom of* N. naja* in Different Geographic Locations

The median lethal dose of venom of* N. naja* following intramuscular injection in the present study was determined to be 0.55mg/kg, 0.66mg/kg, 0.68mg/kg, 0.62 and 0.7mg/kg for NRP, CRP, WRP, SRP, and SARP venoms, respectively.

### 3.4. *N. naja* Venom Induced Pathological Changes in Mouse Organs

Histopathological changes in heart, lungs, kidney, liver, and muscle tissues of mice envenomed with pooled venom from five regions were recorded. However, all region pooled venom sampled had similar pathological effects on heart, lungs, kidney, liver, and muscle tissues.

### 3.5. Histopathological Changes in Skeletal Muscles

Microscopically, myonecrosis and slightly haemorrhagic cells were observed in all the tissues samples including gastrocnemius muscle dissected 1h after injection of venom except SARP and SAP (Table 1). However, severity levels did not increase over time. NRP injected samples demonstrated moderate infiltration of inflammatory cells comparing to other samples. Dense infiltration of inflammatory cells and necrotic cells was recorded in all the tissues samples, which are obtained from the mice sacrificed 3 hours after injecting the venom. But compared to the other tissue sections from other four regions, skeletal muscle tissue obtained from samples was injected with pooled venom of NRP accompanied with high degree in infiltrating inflammatory cells and necrotic cells.

### 3.6. Histopathological Changes in Cardiac Muscles

**Figure 3:**
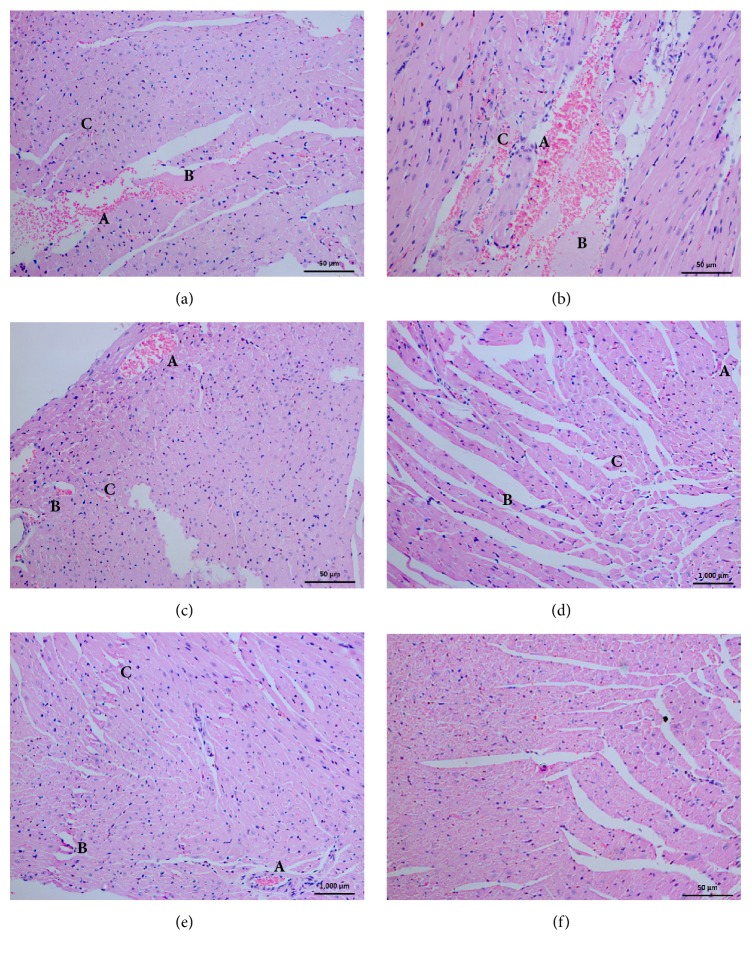
Histopathological changes in cardiac muscles, after 3hr from injection of* N. naja* venom from (a) Western Region Population, (b) Central Region Population, (c) North Region Population, (d) Sabaragamuwa Region Population, (e) Southern Region Population, and (f) Control. (A) Congestion. (B) Necrotic fibers. (C) Inflammations.

According to the histopathological studies on cardiac tissue all the venom samples were found to be highly cardiotoxic (Table 2). The cardiac tissue samples obtained from mice treated with venom consisted primarily of congestion, focal and occasional myonecrosis, and occasional lymphocytic and neutrophilic infiltration (Figure 3). These observations were recorded within 1hr after injection of all the regional venom. Moderate focal and occasional myonecrosis and clumping erythrocytes in the tissues were observed 1hr after in the CRP venom injected samples. Also, within three hours, CRP injected samples exhibited sever damage comparing to other regions. However, comparing the degree of damage on each cardiac tissue sample due to venom from each region, the tissue samples treated with CRP venom associated with high level of necrotic damage than other samples. Despite this difference there were no distinguishable lesions to differentiate the toxic effect of venom from each region on the cardiac tissue. All the tissues samples showed clumping of erythrocytes, which might have been induced by the effect of composite of venom on coagulation cascade causing severe coagulation disorders. The minimum venom dose that caused congestion in heart tissue was 0.2 mg/Kg in NRP, CRP, WRP, and SRP and 0.32 mg /Kg in venom from SARP.

### 3.7. Histopathological Changes in Lung Tissues

**Figure 4:**
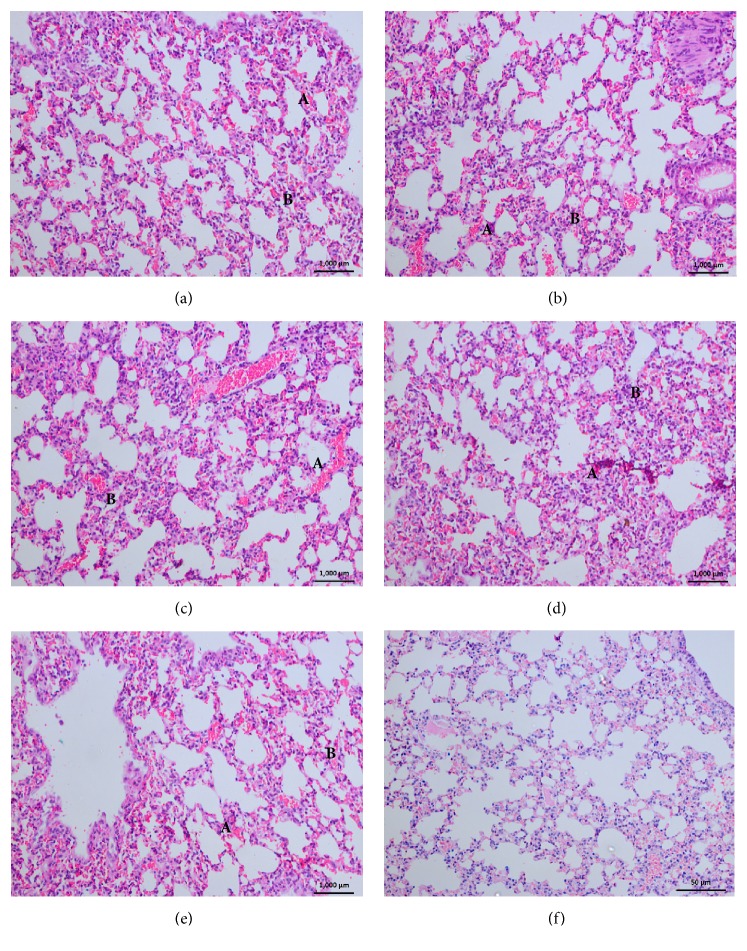
Histopathological changes in lung tissues, after 3hr from injection of* N. naja *venom from (a) Western Region Population, (b) Central Region Population, (c) North Region Population, (d) Sabaragamuwa Region Population, (e) Southern Region Population, and (f) Control. (A) Alveolar haemorrhages. (B) Interstitial septal widening.

All the venom samples from the five distinct regions were found to be causing evident pathological anomalies on pulmonary tissues (Table 3). Infiltration of inflammatory cells into bronchiole lumen was observed microscopically within 1hr after injection of all regional venom samples. Histopathological changes in alveolar septae were accompanied with infiltration of inflammatory cells into interstitial tissue and congestion of capillaries and irregular capillary endothelium, arteritis, and focal anomalies in alveoli epithelium. Alveolar haemorrhage was a general observation associated with lung tissue of all mice envenomed by all venom samples from five regions (Figure 4). However, when compared to the other four regions, SARP venom injected samples did not unfold these pathological anomalies within 1-hour time period. The minimum dose of venom which caused pulmonary congestion was 0.2 mg/Kg for all regional venom samples. Severe inflammatory changes in pulmonary tissue obtained from mice that died within 6 hours following envenoming with pooled venom samples of NRP are the only pathological variation that was found when compared with the others.

### 3.8. Histopathological Changes in Kidney

**Figure 5:**
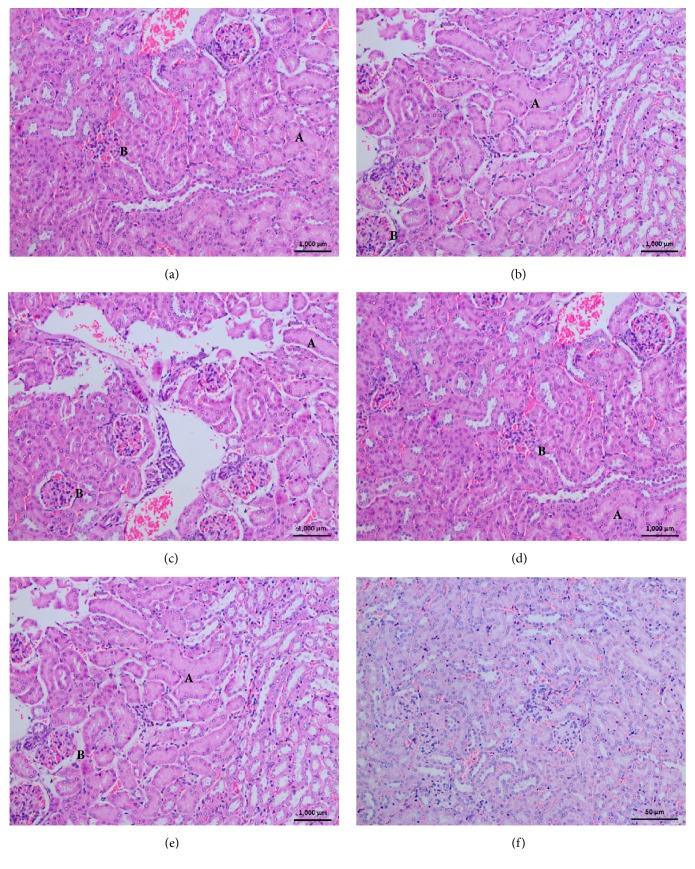
Histopathological changes in kidney, after 3hr from injection of* N. naja *venom from (a) Western Region Population, (b) Central Region Population, (c) North Region Population, (d) Sabaragamuwa Region Population, (e) Southern Region Population, and (f) Control. (A) Acute tubular necrosis. (B) Congestion.

The samples of renal tissue sections from mice, which are injected with cobra venoms from each region, revealed altered histology compared to the control mice group which are given saline (Table 4). Glomeruli of these were observed with degenerative changes such as irregular capsule membrane and congestion of capillaries. Within 1hr NRP and CRP regional venom injected samples were found to have this congestion of glomeruli. Renal tubules undergoing degenerative changes of acute tubular necrosis were observed in all tissue samples. These changes contained tubular vacuolization, necrotic tubular epithelium, capillary congestion, inflammatory cellular infiltrations into renal interstitium, and degeneration of the distal and proximal convoluted tubules (Figure 5). The venom from SARP region did not exhibit any infiltration of inflammatory cells during the 1-hour period. We did not observe any significant differences among the five regional venoms. However, the NRP and CRP pooled venom injected mice which were sacrificed after 6 hours were obtained with renal tissues which showed maximum nephrotoxic effects comparing to the other venom samples from WRP, SARP, and SRP. The minimum venom dose which caused capillary congestion in kidney tissue was 0.3 mg/Kg for venom samples from all the five regions.fv

### 3.9. Histopathological Changes in Liver

**Figure 6:**
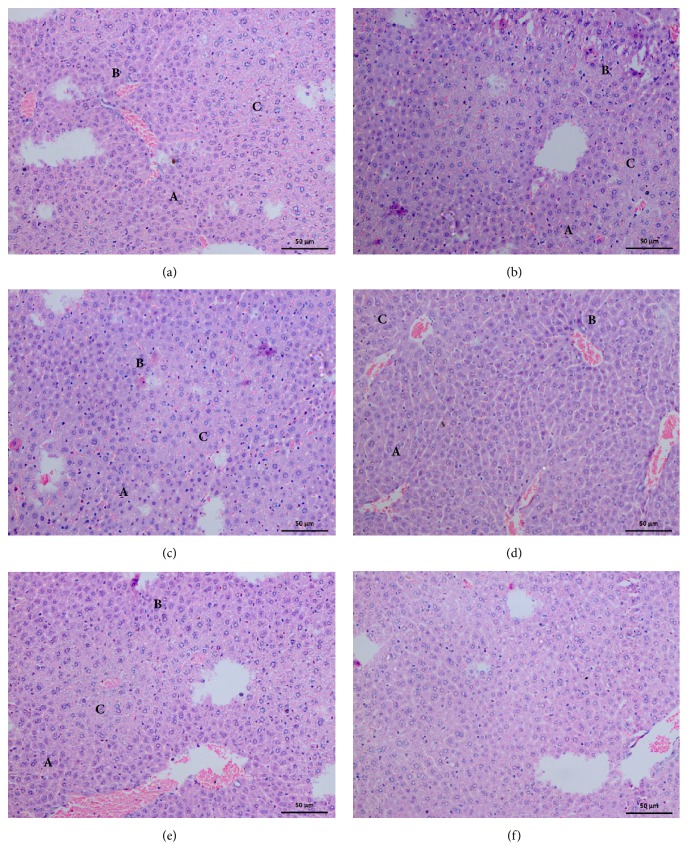
Histopathological changes in liver, after 3hr from injection of* N. naja *venom from (a) Western Region Population, (b) Central Region Population, (c) North Region Population, (d) Sabaragamuwa Region Population, (e) Southern Region Population, and (f) Control. (A) Lytic necrosis. (B) Hepatocyte degeneration. (C) Zonal hepatocellular necrosis.

Microscopically, all the hepatic tissues sections were found with marked changes from their normal histology due to the effects from injected venom regardless to their region (Table 5). All the pathological damage was mildly demonstrated within 1 hour and gradually increased with the time. These microscopic lesions in the hepatic tissues contained both types of hepatocellular necrosis. Out of these two, the zonal hepatocellular necrosis which contains the centrilobular necrosis, random hepatocellular necrosis, focal hepatic necrosis, multifocal hepatic necrosis, and occasional hepatic necrosis was primarily observed in the liver tissue (Figure 6). Other than these necrotic changes, infiltration of inflammatory cells and congestion of blood vessels were observed as general inflammatory changes. No haemorrhages were observed macroscopically or microscopically as a pronounced lesion in any of the hepatic tissue samples collected at any time period. When comparing and contrasting the injurious insult to the hepatic tissue according to the region of venom collection, microscopic lesions in liver samples given with venom from SARP were more prominent than other four regions.

### 3.10. Enzymes Activities

**Figure 7:**
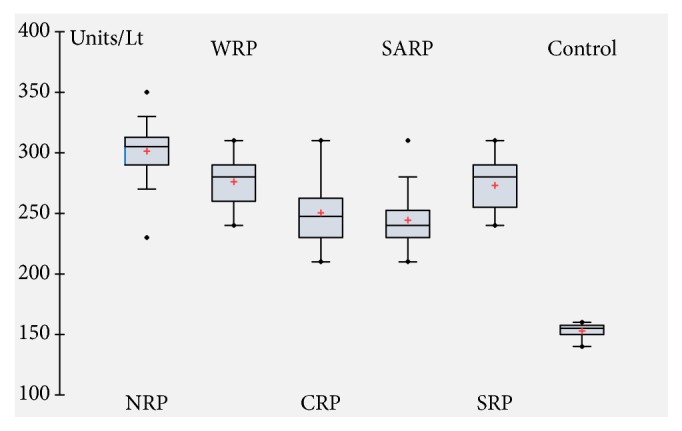
Serum glutamate-oxaloacetate transaminase (SGOT) activity.

**Figure 8:**
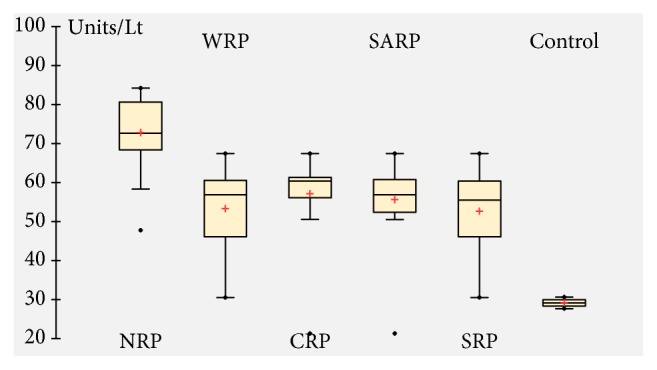
Serum glutamate-pyruvate transaminase (SGPT) activity.

**Figure 9:**
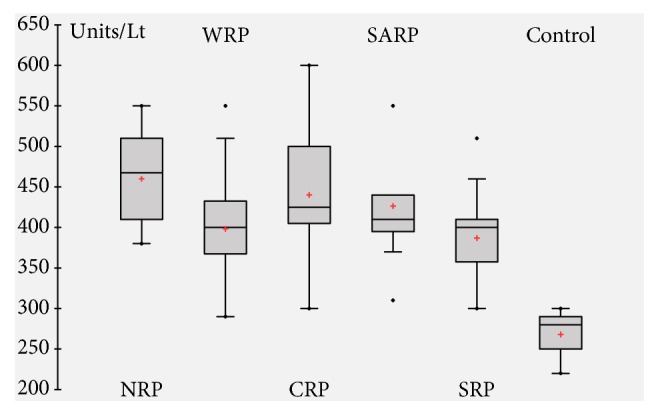
Serum Creatine Kinase (CK) activity.

A significant increase in serum SGOT (Figure 7), SGPT (Figure 8), and CK (Figure 9) activities was observed after 1 hour of venom injection, compared with the control mice group. When comparing the SGOT activity between regional venom injected mouse groups significant difference was observed in the values obtained (*F = *15.37065*; p = *0.00001). SGPT activity also revealed significant difference between regional venom injected mouse groups (*F = *14.51459*; *p = 0.00001) and CK activity between regional venom injected mouse groups was recognized to have a significant variation in their values (*F = *4.92769*; p = *0.001182).

## 4. Discussion

Despite the variation in venom between different species, biochemical composition and pathogenesis due to envenomation by a particular species might vary according to their geographical locations [16, 19–25, 50]. In the present study we investigated the applicability of this theory to the* N. naja* species found in Sri Lanka declared as one of the most venomous snakes in the country. Primary focus of this study arrayed to identify the systemic and biochemical pathology induced by* N. naja* and understands whether this exerted toxic pathology on different organs of mice differentiated according to the geographical location of the particular* N. naja*.

In this study, we discovered that the LD_50_ values obtained for* N. naja* venom injected via IM routes are different from each value. Average LD_50_ value (IM) obtained from lethality studies conducted using venom of* N. naja* originated from five distinct regions demonstrated lower LD_50_ values. When this lethal dose of* N. naja* of Sri Lanka is compared to the previously recorded LD_50_ values of Indian* N. naja* [51], we can conclude that Sri Lankan* N. naja* is more toxic than the* N. naja* in India, whereas intraregional variation of the LD_50_ values resulting from venom of each region did not have any significant difference. This might be due to the less land mass in Sri Lanka when comparing to India. However, the NRP pooled venom sample holding the lowest LD_50_ value was recognized as the most toxic venom out of all five regions. Our results of this study concord with several other studies which have revealed that the lethality of the snake venom varies from species to species and even within the same species according to their geographic origin [15, 35]. The differences in venom lethal toxicity between India and Sri Lanka were identified to be considerably notable. This can be the reason for the considerable variation in the clinical situation of human envenomation in Sri Lanka when compared to India [6]. However, advanced proteomics studies are needed to be conducted to identify this variation related to identifying possibly influencing clinical symptoms and signs following envenomation.

In this study the pathological effect of each regional venom sample according to the time of venom administration was evaluated and the pathological data witnessed a toxic effect exerted within 1 hour after IM injection regardless to their originated location. Similar to previous records on relationship between effects of envenomation with the time of envenomation [52] current study also found severity of the venom increasing parallel to the time.

Numerous studies indicate that cobra venom can induce profuse pathological anomalies in vital tissue structures [12, 13, 18, 51, 53]. Histopathological tissue sections of gastrocnemius muscle, heart, kidney, liver, and lungs of mice injected with venom from all five regions manifested severe pathological anomalies including tissue necrosis associated with haemorrhage and congestion, while control mice group resulted in their normal tissue structures. Several studies with similar histopathology in mice tissues suggest that the potent toxic effect of nonenzymatic fraction of cytotoxins and phospholipase A_2_ (PLA_2_) enzyme leads to these extensive necrotic observations in venom affected tissues by the formation of pores and disturbing the phospholipid structure of lipid bilayer [12, 16, 54, 55]. Other research reports state similar haemorrhages and congestion in several tissue types as haemostatic disturbances produced by* N. naja* venom components of metalloproteinases and PLA_2_ [13, 35, 56]. Therefore, we can conclude that venom of Sri Lankan* N. naja* contains functionally similar components proceeding to damage the vessel endothelium and extracellular matrix causing blood leakage into tissues.

All the vital organs and the skeletal muscle tissues acquired from the envenomed five regional mice groups demonstrated the basic defensive response of severe inflammatory cell infiltration, a reaction identified as a primary tissue activity of envenomed animals against the injurious effects of PLA_2_ and PLA_2_ generated reactive oxygen species of cobra venom [35, 56, 57]. A study on Indian* N. naja* found with similar leukocyte recruitment in tissues documents that PLA_2_ content in cobra venom varies according to their geographical location and this automatically changes the ability of attracting inflammatory cells [35]. But in our study, we did not recognize any significant difference of this inflammatory change according to their region of venom collection.

While histopathological studies on skeletal muscles of venom injected mice revealed widespread cellular pathology, control group contained the fine structural aspect of skeletal muscle except to the physical damage done due to injection. In comparison, skeletal muscles injected with NRP pooled venom sample demonstrated severe myonecrosis which appeared quickly than other four regions suggesting possible intraregional variation in composition and toxic effect in the venom of Sri Lankan* N. naja*. Various other studies indicate that the amount of cytotoxins and PLA_2_ might vary according to the location of origin of the particular cobra [35]. The fine sarcolemma membrane disruption by the above toxins in cobra venom results in abnormal Ca_2_^+^ influx inducing various types of cellular pathology [35, 55].

Envenomation of* N. naja* is well known for its effect induced on the cardiac tissue by the cardiotoxin fraction of low molecular polypeptides in their venom [58]. The cardiotoxins are rapidly absorbed, circulate in blood stream, combine with the enzyme PLA_2_, and initiate the destruction of membrane structure of cardiac myocytes. The ultimate result of this activity is the myotoxicity induced cellular pathology including necrotic, inflammatory, and congestive changes in cardiac tissue similar to the current study. All the tissues samples were found with clumping of erythrocytes, which might have been induced by the effect of composite of venom on coagulation cascade causing severe coagulation disorders. Clumped erythrocytes observed are also caused due to the direct lytic activity of cardiotoxins. When comparing the severity level of these pathological alterations in accordance with the venom collected region, CRP venom injected mice contained severe changes in cardiac tissue than others. According to previous reports this intrazonal diversity among pathological alterations may originate due to quantitative variation of potential toxins basically differentiating with the specific location of the responsible cobra [35].

Majority of deaths caused by the* N. naja* envenomation occur due to asphyxia, the subsequent result of post- and presynaptic neurotoxin activity on nerve transmission. The pulmonary tissue of all regional envenomed mice contained the vascular anomalies mediated by cobra venom metalloproteinases, the capillary congestion, irregular capillary endothelium, arteritis, and alveolar haemorrhage. Some studies suggest that cobra venom PLA_2_ mediates the activity of infiltration of inflammatory cells into bronchiolar lumen and the alveolar septae [16]. Therefore, the leukocytic accumulation causing alveolar septal blockage and the disrupted alveolar epithelium, in this study, demonstrate the possible pathological effect of PLA_2_ in Sri Lankan cobra venom on pulmonary tissue of mice.

The postsynaptic cytotoxins find in elapid venom is well known for its easy accumulation in kidney tissue [13]. Excretion of most toxic substances in snake venom usually occurs via the kidneys of the envenomed animal [55, 59]. As cobra venom is elapid venom, cobra venom induced nephrotoxicity can also be applied with the above-mentioned mechanisms. Present study revealed the similar pathological lesions in kidneys related to acute renal failure, which has been categorized by previous studies about nephrotoxic effect of* N. naja* venom [13, 53]. The fine structures of kidneys were disrupted due to the visible histopathological glomerular and tubular lesions. Tubular injury contained the typical degenerative changes of acute tubular necrosis. Many studies indicate cytotoxins and PLA_2_ as the mediators of venom induced nephropathy [13, 53]. Therefore, renal lesions of this study contribute towards understanding inflammatory and necrotic activity of these components in Sri Lankan* N. naja* venom. A noticeable variation among above lesions in kidney tissues of envenomed mice according to their geographical location was not found.

Since detoxification of any toxic component is one of the primary actions of liver, most of the toxic components get the opportunity to exert their pathological activities directly onto the liver parenchyma. The hepatic tissue of the mice administered with venom from all five regions in this study witnessed this fact by manifesting variable degree of cellular swelling and cytoplasmic changes attributed with other pathological alterations leading to massive hepatocellular necrosis than other organs. Many studies with similar observations unveil cobra venom PLA_2_ as the etiology for this hepatic damage [12, 53]. The phagocytic cell migration induced by the cobra venom observed in the current study also disrupted the cell structure of hepatic tissue. Elevated amounts of ALT and ALP recorded in blood serum analysis of all five regional mice groups resembled the biochemical evidence for the venom induced hepatocellular dysfunction. When this serum analytical data combines with the current observations in histopathology of liver tissue, venom from Sabaragamuwa province was determined as the least hepatotoxicity induced venom.

Creatinine kinase serves as blood serum indicator of tissue destructions associated with skeletal and cardiac muscles and possible renal dysfunction [35, 53]. Therefore, the inflated levels of CK in blood serum of all envenomed mice regardless to their region appoint the toxic involvement of Sri Lankan* N. naja* with the above-mentioned tissues. The highest values of CK were obtained from the venom of NRP and CRP. When combining the histological variation results of myotoxicity on skeletal and cardiac muscles with this biochemical result, NRP and CRP pooled venom can be concluded to own the highest myotoxicity.

## 5. Conclusion

The histological and serum biochemical data of this study contributed towards revealing the specific pathological effect of* N. naja* venom on rodent vital organs and skeletal muscle tissue and this illustration supports identification of possible toxic effect on any envenomed animal by* N. naja* in Sri Lanka. The present study also demonstrated some fluctuations in these pathological alterations providing the baseline information for the possible intraspecies variation among Sri Lankan* N. naja* venom according to their location of origin. When comparing these variations of Sri Lankan* N. naja* with the observations recorded for Indian* N. naja,* this intraspecies variability in pathology of cobra venom related to their geography demonstrated a significantly lower level than Indian* N. naja*. This might be due to the less land mass in Sri Lanka when compared with the states of India. Even though the recorded variability among regional histopathology is slight, this investigation provides the basic evidence for the qualitative and quantitative variation of the composition of Sri Lankan* N. naja* venom based on their location of origin. Further, proteomics and other biochemical studies should be conducted to determine the intraregional variation in the venom composition and their exact mode of action on different organs.

## Figures and Tables

**Table 1:** Major histopathological changes in skeletal muscles induced by *N. naja* venom from distinct geographic origins in Sri Lanka. Score: 0: absent; +: mild; ++; moderate; and +++: severe [45].

Time since being envenomed (h)	1					3					6				
Geographic Locations	NRP	CRP	SARP	WRP	SAP	NRP	CRP	SARP	WRP	SAP	NRP	CRP	SARP	WRP	SAP
Myonecrosis	++	+	+	+	+	++	++	++	++	+	++	++	++	++	++

Haemorrhagic cells	+	+	0	+	0	+	+	+	+	+	++	+	+	+	+

Infiltration of inflammatory cells	++	+	+	+	+	+++	++	++	++	+	+++	++	++	++	++

Necrotic cells	++	++	++	++	++	+++	++	++	++	++	+++	++	++	+++	++

**Table 2:** Major histopathological changes in cardiac muscles induced by *N. naja* venom from distinct geographic origins in Sri Lanka. Score: 0: absent; +: mild; ++; moderate; and +++: severe [45].

Time since being envenomed (h)	1					3					6				
Geographic Locations	NRP	CRP	SARP	WRP	SAP	NRP	CRP	SARP	WRP	SAP	NRP	CRP	SARP	WRP	SAP
Congestion	+	+	+	+	+	+	++	+	+	+	++	+++	++	++	++

Focal and Occasional Myonecrosis	+	++	+	+	+	++	+++	++	++	++	+++	+++	+++	+++	+++

Infiltration of inflammatory cells	+	+	+	+	+	+	++	++	++	+	+++	+++	++	++	++

Clumping of Erythrocytes	+	++	+	+	+	++	+++	++	++	+	++	+++	++	++	++

**Table 3:** Major histopathological changes in lung tissues by *N. naja* venom from distinct geographic origins in Sri Lanka. Score: 0: absent; +: mild; ++; moderate; and +++: severe [45].

Time since being envenomed (h)	1					3					6				
Geographic Locations	NRP	CRP	SARP	WRP	SAP	NRP	CRP	SARP	WRP	SAP	NRP	CRP	SARP	WRP	SAP
Congestion	+	+	0	+	+	++	+	+	++	+	++	++	++	++	++

Infiltration of inflammatory cells	+	+	+	+	+	++	+	+	+	+	+++	++	++	++	++

Haemorrhagic cells	+	+	0	+	+	+	+	+	+	+	++	++	++	++	++

**Table 4:** Major histopathological changes in Kidney by *N. naja* venom from distinct geographic origins in Sri Lanka. Score: 0: absent; +: mild; ++; moderate; and +++: severe [45].

Time since being envenomed (h)	1					3					6				
Geographic Locations	NRP	CRP	SARP	WRP	SAP	NRP	CRP	SARP	WRP	SAP	NRP	CRP	SARP	WRP	SAP
Congestion of glomeruli	+	+	0	0	0	++	++	+	+	+	+++	++	++	+	+

Acute tubular necrosis	+	+	0	0	0	++	++	+	+	+	+++	++	+	+	+

Capillary congestion	+	+	+	+	+	++	++	+	+	+	++	++	+	+	+

Infiltration of inflammatory cells	+	+	0	+	+	++	+	+	++	+	++	++	+	+	+

**Table 5:** Major histopathological changes in liver by *N. naja* venom from distinct geographic origins in Sri Lanka. Score: 0: absent; +: mild; ++; moderate; and +++: severe [45].

Time since being envenomed (h)	1					3					6				
Geographic Locations	NRP	CRP	SARP	WRP	SAP	NRP	CRP	SARP	WRP	SAP	NRP	CRP	SARP	WRP	SAP
Clumping of erythrocytes in blood vessel	+	+	+	+	+	++	++	++	++	++	++	++	+++	++	++

Zonal hepatocellular necrosis	+	+	+	+	+	++	++	++	++	++	+++	++	++	++	++

Infiltration of inflammatory cells	+	+	+	+	+	++	++	++	++	++	++	++	+++	++	++

Congestion	+	+	+	+	+	++	++	++	++	++	++	++	++	++	++

Centrilobular Necrosis	+	+	+	+	+	++	++	++	++	++	++	++	++	++	++

## Data Availability

The data used to support the findings of this study are available from the corresponding author upon request.
